# ‘If you are feeling alone and you are not feeling safe, it impacts everything’: a mixed-methods exploration of international students’ accommodation, subjective wellbeing and mental health help-seeking

**DOI:** 10.1186/s12889-024-18691-8

**Published:** 2024-05-08

**Authors:** Tim Corney, Karin du Plessis, Brett Woods, Catherine Lou, Anita Dewhurst, Daveena Mawren

**Affiliations:** 1 Institute for Sustainable Industries and Liveable Cities, Victoria University, Melbourne, Australia; 2 Centre for Forensic Behavioural Science, Swinburne University, Alphington, Australia

**Keywords:** International Education, Foreign students, Student welfare, Wellbeing

## Abstract

**Background:**

The international education sector is important not only to Australian society, but also to the national economy. There are growing concerns about the potential wellbeing challenges facing international students in their host country, owing to acculturative stress; including loneliness, isolation and experiences of racism. Risks include poor mental health and decreased likelihood to access support due to stigma, language and cultural barriers, not knowing where to seek help, and poor mental health knowledge.

**Methods:**

This study explored students’ perceptions of their accommodation, subjective wellbeing (through the Personal Wellbeing Index, [‘PWI’]), mental health help-seeking and individual engagement with evidence-based everyday health promotion actions (informed by the ‘5 Ways to Wellbeing’ model) through an online survey (*N* = 375) and three online focus groups (*N* = 19). A mixed-methods approach using descriptive statistics, ANOVA, regression analysis and thematic analysis, were used.

**Results:**

The PWI of international students in the survey was observed to be substantially lower (*M* = 60.7) than that reported for the Australian population (*M* = 77.5). Accommodation impacted on wellbeing (loneliness, belonging, connectedness) in a number of different ways including through location, safety, and shared accommodation. In terms of help-seeking, international students noted a number of barriers to accessing support for mental health: cost of accessing support, language and cultural barriers, lack of information on where to find support and stigma.

**Conclusions:**

In the discussion, implications of the findings are considered, including that more could be done to shape policy and practice in service and facility provision around wellbeing, connectedness, and help-seeking for mental health support of international students.

**Supplementary Information:**

The online version contains supplementary material available at 10.1186/s12889-024-18691-8.

## Background

The international education sector is important not only to Australian society, but also to the national economy. With over 200,000 international students from 170 countries in 2018, the State Government of Victoria in Australia classified international education as a multi-billion-dollar export earner [1]. International students form a significant population set in the Australian community, but there are growing concerns about the potential wellbeing challenges they face, which are not only different but also more pronounced from those experienced by domestic students [2, 3]. This life transition period for international students includes taking on new responsibilities, sourcing accommodation, independent money management, balancing work, study and private life, as well as adapting to, and succeeding in, tertiary education [4]. These adaptation and acculturation tasks are made more complex by transitioning to independent living in a foreign country far away from familiar family and community networks, the grief and loss associated with that transition, as well as dealing with cultural and language differences, including studying in English (often as a second language) at a tertiary academic level. While this acculturative stress is recognised as temporary, because most international students plan on returning home at completion of their studies (i.e., this is different from a permanent migrant experience), and it varies by country and individual student experience, it can be overwhelming, and may contribute to poor mental health [4], particularly if exacerbated by loneliness, isolation and experiences of racism [5, 6]. University students are often at an age (i.e., 17–25 years old) when mental illness can emerge − 75% of mental illness onsets before the age of 24 [7]. International students are at particular risk for experiencing poor mental health (due to the abovementioned stressors), and less likely to access support due to stigma, language and cultural barriers, not knowing where to seek help, poor mental health knowledge, and self-limiting help-seeking attitudes (e.g., believing that support services are only for acute mental health issues) [2, 3, 5]. An Australian study found that Chinese international students’ levels of stress and anxiety were significantly higher than their Australian counterparts [8]. This risk for poor mental health is further illustrated in a recent report by the Victorian State Coroner who examined the circumstances of 27 international student suicides as well as a cohort of suicides among Australian-born students [6]. It found a lower prevalence of diagnosed mental illness amongst international students (15% vs. 67%), and that fewer international students sought support for mental health issues compared to Australian-born students (22% vs. 57%). The report concluded that there are underlying systemic issues in engaging international students in mental health treatment.

Wellbeing is a multifaceted construct that includes meeting basic psychological needs (e.g., autonomy, competence, relatedness) as well as other factors such as satisfaction, resilience and balance [9]. For international students this is broader than their study context and includes the interplay between multiple systems (e.g., individual, social, home environment, educational institution, cultural, community etc.) [9]. Previous research indicates that social disconnectedness, isolation, lack of belonging and loneliness also impact on international students’ mental health and wellbeing [10, 11]. Social relationships are seen as a protective factor in both prevention and recovery of poor mental health, and this can be particularly true when people experience a loss of their social identity, for example through a major life transition such as becoming an international student in a foreign country [12]. Peer support is recognised as a valuable alternative to immediate or extended family for students [11, 13], which in addition to provision of social connection helps students to organically gain an understanding of coping strategies and more successfully transition into university life [14, 15]. Connectedness became more challenging for international students who remained in Australia during the COVID-19 pandemic when they had to self-isolate, study online instead of in classrooms, and were often stranded in their host country without access to welfare support [16].

For international students, social connections and networks are often formed through a variety of common factors, such as their university course, workplace, home/accommodation location, culture, religion, recreational pursuits and personal interests/hobbies [17]. In addition to connections forming organically, research highlight the importance of these social connections being intentionally designed, facilitated and encouraged by tertiary education stakeholders (e.g., universities, residential colleges/student accommodation, student membership bodies, and community/social groups) to enhance belonging, overall wellbeing and relational wellbeing, and in particular to enable culturally appropriate connections for international students who are more at-risk of isolation and loneliness [15, 16, 18–21].

Research from the United Kingdom indicates that for students in university accommodation who feel uncomfortable and have a low sense of belonging, there is an association with higher levels of depression, anxiety, and loneliness [22]. A recent qualitative Australian study found that one of the significant mental health and wellbeing challenges for international students (in addition to academic stressors, mental health issues, lack of social connection, and financial pressures), was accommodation concerns [3]. It highlighted costs, proximity, difficulties with housemates, as well as exploitation and discrimination by landlords, as notable issues for international students [3]. As in other countries, accommodation costs and housing affordability are a particular issue for Australian international students, who often have to share accommodation with other students at the expense of privacy, often sharing bedrooms to save money, and this in turn impacts wellbeing [23]. Conversely, it can be noted that a more recent accommodation trend is the emergence of high-end purpose-built all-inclusive lifestyle residential communities which are often managed by private/commercial companies. Marketing of these might be particularly targeted to affluent international students, which some authors caution could be generating geographies of exclusion and segregation between the students and local communities, as well as within the student population [24].

Informal, everyday individual strategies may offer utility for wellbeing with international students, in light of the reported challenges in engaging this population in formal mental health supports [6]. The ‘5 Ways to Wellbeing’ [‘5 Ways’] approach was developed for the UK Government to provide an evidence-based set of everyday actions to enhance personal wellbeing [25, 30]. It seeks to promote a holistic health perspective including a focus on emotional, social, spiritual and psychological wellbeing [25, 30], and includes the following five “generic” [25, p. 3] areas of action for individuals to undertake to improve their wellbeing: ‘connecting with others’; ‘being physically active’; ‘learning new things’; ‘being mindful’; and ‘helping others’. Farrier et al. [26, p. 72] state that 5 Ways has ‘become one of the most widely used evidence-informed frameworks’ in wellbeing promotion. The model has been employed in health promotion initiatives internationally with diverse populations [26, 27], and has been adopted in Australian and New Zealand health and hospital contexts and in some Australian universities.

The study aimed to obtain a snapshot of the wellbeing of international students living in Melbourne, Victoria, so as to identify predictors of wellbeing, areas of risk to wellbeing and levels of engagement in strategies commonly associated with increased wellbeing and circumstances of this population. It also examined perceptions of stigma, help-seeking and access/barriers to support for mental health issues. The study data underlies further knowledge translation, advocacy and health promotion activities to university and student leaders and stakeholders, including through the health and wellbeing promotion activities identified (not reported here).

It should be noted that the study was conducted in 2020 in the early period of the pandemic, when lockdowns were being initiated in Victoria, Australia. This necessitated moving data gathering online. The survey instrument directed participants to answer the questions relating to the 5 Ways actions based on how they *usually* engaged in the actions, inviting them to consider their activities prior to COVID-19 restrictions.

## Methods

Prior to conducting the study institutional ethics approval was completed (HRE20-047) and data was collected, recorded and stored in accordance with the university’s research ethics and consent criteria. First, a contextual focus group with international student association leaders and one survey pilot focus group (with international students recruited through international student associations) were conducted to inform development and design of the online survey and focus group interview schedule.

As the study gathered quantitative and qualitative data through the online survey and online focus group interviews, the researchers used a mixed-method research approach for complementarity and convergence of data [28]. In part, the results from the survey informed the development of the focus group questions (sequential design). The focus groups were also used to obtain an understanding of the larger context of the survey and more in-depth understanding the social reality of international students (e.g., through examples of their lived experience) [28].

Participants for the survey were recruited through six State-wide international student associations. These international student associations emailed the invitation to participate to their membership networks and shared the study on their social media accounts. Focus group participants were recruited through the six international student associations. Students self-selected to participate. International students from all eight public universities and Technical and Further Education (TAFE) Colleges in Victoria, Australia were invited and participated in the study.

In Victoria, Australia, the most dominant international student cohort is from China (21%), and thus we offered the online survey in both English and Mandarin. The English survey was translated into Mandarin by a professional translator and reviewed by a member of the research team (whose first language is Mandarin) to ensure there was similar meaning across the survey in both languages. The survey asked a number of demographic questions and measured subjective wellbeing on the Personal Wellbeing Index [‘PWI’] [29]. In choosing this instrument consideration was given to the strengths and weaknesses of self-report in measuring subjective wellbeing [30, 31] as well as the strong psychometric properties of the PWI [29]. The PWI was developed by the Australian Centre on Quality of Life and is an internationally recognised, evidenced-based measure which indexes subjective wellbeing through life domains around standard of living, health, achieving in life, relationships, safety, community-connectedness, future security, and religion and spirituality [29, 32]. Owing to the “flexibility, resonance and widespread appeal of [the] 5 Ways” framework [26, p. 71] for wellbeing promotion with diverse groups [33, 34], the researchers included questions relating to participants’ perceptions of the importance of each of the 5 Ways actions (‘connecting with others’, ‘being physically active’, ‘learning new things’, ‘being mindful’ and ‘helping others’) and the frequency in which they engage in these actions. Participants were additionally asked to describe the ways in which they undertake these actions and ideas for how organisations (such as universities, community groups or student groups) might enable international students to engage in these actions more often (results not reported here). The survey also included bespoke items exploring student experiences with accommodation (such as perceived safety of the residence and issues with flatmates) as well as perceived barriers to accessing support for wellbeing and health (such as stigma from family and friends).

Separate to the surveys, three semi-structured focus groups (*N* = 19) were conducted online (during COVID-19 lockdown restrictions) by members of the research team in groups of five to seven students, recruited opportunistically, based on availability and interest [35] through international student associations. Each of the focus groups took approximately 90 min; two were conducted in English, and one in Mandarin. The English focus groups were facilitated by the same researchers; the Mandarin focus group was facilitated by a member of the research team who is a native Mandarin speaker and who had attended the English focus groups, ensuring consistency. Participants were asked a series of questions on the topic of health and wellbeing (see supplementary materials for focus group questions), and responses generated data both individually and through interaction between participants and the interviewer. Tentative themes were presented to focus group participants in situ for further comment and confirmation [36].

Focus group interviews were audio recorded and transcribed verbatim - the transcription was provided by a professional service, and the Mandarin transcript translated by a professional translator. The data (audio-recordings, transcripts and researcher notes) were analysed by the research team using thematic analysis [37] involving identification of dominant patterns and themes in the responses. Both deductive and inductive techniques [36] were used which enabled the capture of naturally emerging themes from the data as well as key themes that aligned with the research questions. As the analysis sought to answer the research questions, the questions themselves were a useful lens to guide the deductive component of the analysis [38]. Themes were cross-checked with field-notes, and discussions about emerging themes were held by the research team and themes were refined. Themes were subsequently fed back to a smaller group of focus group participants (who had participated in the earlier focus groups) (convenience sample) for authentication, validation, comment and review. The final selection of themes and illustrative quotes were based on relevance to the aims of the study [37]. Three levels of data collection and analysis (individual, group and interactive) enabled depth and reliability [39].

The statistical analysis was conducted in SPSS version 25. Patterns in responses to the individual survey items were explored by obtaining descriptive statistics such as means, standard deviations and frequencies. Specifically, it should be noted that for comparative purposes the Personal Wellbeing Index (PWI) scores were calculated by the aggregate of individual scores, rather than by using survey mean scores (both are standard practice). A series of one-way ANOVA tests (see supplementary material) were conducted to assess for significant differences in responses across demographic characteristics, living situations (including accommodation issues such as conflict with flatmates or feeling unsafe and perceived barriers to accessing support for wellbeing and health. Significant variables identified in the ANOVA were entered into a regression model (see Table 3), along with variables assessing frequency of engagement with each of the 5 Ways to Wellbeing strategies, in order to identify significant predictors of wellbeing.

At conclusion of the quantitative and qualitative analyses, the data were integrated by the research team to present a snapshot of the lived experiences of international students living in Melbourne, Victoria. Illustrative quotes are used here to exemplify key qualitative themes and integrate with quantitative findings.

## Results

The demographic characteristics of these international students are presented in Table 1. While a total of 632 surveys were returned, missing data (> 30% of survey items incomplete) necessitated exclusion of 257 cases. Chi-square analyses indicated no significant difference between completers and non-completers across age *X*^*2*^ (1) = 0.638, *p* > .05), or University attended (*X*^*2*^ = 0.260,*p* > .05) or language used for survey completion (*X*^*2*^ (1) = 2.122,*p* > .05). Of the 375 participants, 305 (81%) completed the survey in English, and 70 (19%) completed it in Mandarin.

Almost two thirds (*n* = 220, 59%) of the international students in this sample were 18–25 years old, with the remainder older than 25 years (*n* = 155, 41%). Over half (*n* = 238, 63.5%) of the participants identified as female. The international students studying in Victoria who participated in the survey were from 43 different countries. Almost a quarter of the students were from China (*n* = 99, 27%), 16% (*n* = 59) from India, and 13% (*n* = 48) from Nepal. 27% (*n* = 101) of students were from 13 other Asian countries. Of these, 19% (*n* = 72) were from South East Asian countries including Vietnam, Indonesia, Malaysia and the Philippines, and 8% (*n* = 29) from other countries in the South Asian continent including Sri Lanka, Bangladesh and Pakistan. Over a third of the students had been in Australia for one year or less (*n* = 148, 40%). Two thirds were enrolled in a university degree (37% in an undergraduate degree, and 36% in a post-graduate degree, the remainder studying a variety of courses).

**Table 1:** Demographic characteristics of survey sample (*N* = 375)

	*n*(%)		*n*(%)
Age cohort		^Country of Birth	
18–25 years of age	220 (58.7)	China	99 (26.6)
25 years+	155 (41.3)	India	59 (15.9)
^+^Gender		Nepal	48 (12.9)
Female	238 (63.5)	Other Asian countries	101(26.9)
Male	128 (34.3)	European country	16 (4.3)
Non-binary, agender, gender diverse	3 (0.8)		
Prefer not to say	4 (1.1)	Middle Eastern countries	16 (4.3)
*Current education level		South American countries	20 (5.3)
Technical and Further Education (TAFE) College	62 (16.7)	Other	9 (2.4)
Undergraduate (University)	138 (37.1)	Primary language**	
Post-graduate (University)	135 (36.3)	English	65 (17.3)
English language course	13 (3.5)	Nepalese	44 (11.7)
Other	24 (6.4)	Mandarin, Cantonese or dialect	105 (28.0)
Years in Australia		Hindi, Punjabi or dialect	45 (12.0)
Less than 1 year	80 (21.3)	Vietnamese	14 (3.7)
1 year	68 (18.1)	Other***	102 (27.2)
2 years	104 (27.7)		
3 years	65 (17.3)		
4 years or more	58 (15.5)		

^+^*n* = 2 missing, *n = 3 missing, ^*n* = 7 missing

**39 different primary languages were represented in the survey sample

***The ‘other’ category included 29 different languages, the largest of which included Spanish, Indonesian, Urdu and Arabic

Compared to data previously observed for the general Australian population [32], the study finds that the overall PWI of international students was substantially lower (*M* = 60.7, *SD* = 19.3) than that of the general Australian population (*M* = 77.5, *SD* = 17.0), and also compared to Australian age cohort norms (18–25 years old, *M* = 75.7, *SD*, 1.8; and 26–35 years old, *M* = 75, *SD* = 0.9) (Table 2). Previous research indicates that generally individuals from Asian backgrounds score lower on the PWI due to cultural response bias, however, the overall PWI score (60.7%) observed for these international students were also lower than the overall PWI score for people in Hong Kong (65.9%) [40], urban China (67.1%) [41], or that found in another collectivist culture, India (74.4%) [42]. Overall PWI scores in the study also differed across country of birth for students from Nepal who had lower wellbeing scores (*M* = 53.5, *SD* = 20.4), than students from China (*M* = 63.6, *SD* = 17.5), India (*M* = 65.9, *SD* = 18.2) or other Asian countries (*M* = 63.3, *SD* = 19.5).

**Table 2:** Descriptive statistics of Personal Wellbeing Index (PWI) items

	M(SD)	M(SD) (Comparative norms*)
1. Standard of living	60.7 (23.9)	78.1 (16.9)
2. Health	66.9 (20.3)	74.5 (19.6)
3. Achieving in life	58.7 (23.0)	73.4 (18.6)
4. Personal relationships	62.0 (26.1)	79.4 (21.3)
5. Personal safety	66.8 (23.6)	79.6 (17.6)
6. Community connectedness	56.0 (24.8)	71.2 (19.7)
7. Future security	54.1 (26.0)	71.2 (19.8)
Overall PWI wellbeing score Comparative PWI norm (18-25) Comparative PWI norm (26-35)	60.7 (19.3)	77.5 (17.0) 75.7 (1.8)^ 75.0 (0.9)^

*Observed comparative norms (32); ^Age comparative PWI norms calculated by survey means method (32)

The focus groups highlighted the important link between accommodation and wellbeing for students. In particular, ‘safety’ was a priority for international students and their families. This included feeling safe, physical safety (e.g., low crime rates, lack of transport, dark/empty streets) and the type of accommodation (e.g., the perception that apartments are safer when not on the ground floor, the importance of accommodation having secure access such as key/entry card, and that there are neighbours around). One quote demonstrates this: *“I feel safety is key. It’s a priority for me and should be for everyone else. I believe that if you don’t feel safe in your own home, it’ll be really difficult to cope with it, because you’ll always be afraid, and your home is literally where you spend most of the time”.* From the survey more than 15% (*n* = 66, 15.3%) students reported feeling unsafe in their accommodation. ‘Location’ also impacted on perceptions of safety, with focus group participants indicating that international students spend considerable time researching accommodation locations and crime rates prior to making choices. There was a perception with international students that city and inner suburb locations were safer, and that living close to friends can help students to feel safe. Living close to their cultural community was important for some students. Access to campus, amenities and transport, as well as distance to study and work, also impacted international students’ accommodation location choices.

The survey showed that the majority of the students (*n* = 331, 77%) shared accommodation with other people, which included a quarter of students (*n* = 113, 26%) who shared a bedroom with another person/people. Two thirds of the international students reported that the cost of accommodation had a significant impact on their wellbeing (*n* = 247, 66%) and for almost one third (*n* = 132, 31%) the people they lived with had a negative impact on their wellbeing. ‘Living with others’ such as housemates provided some students in the focus groups with positive social connections, but for the majority of focus group participant’s housemates negatively impacted their wellbeing due to conflict, cultural and schedule differences. For example one said *“.living with friends that you are not close with, or a stranger, is - has been, quite challenging to my mental health… I would prefer to stay in my room and not go into the common area. You don’t feel like you have your own space. Your own space is the tiny little room”*. Sharing a room with others provided additional challenges, amplifying conflict, cultural and schedule differences, and lack of privacy was a core issue. One participant said *“my room partner, she used to come [home at] any time. It can affect a lot, and it doesn’t matter if I am studying or something like that, and I can’t deny [her access] because she shares the same room… And sometimes when I do need to sleep or something like that. she’d put on music”*. The preference for many of the participants was to live alone, but the cost was prohibitive. Cost also impacted choice as did housing instability, for example one participant noted: *“It was difficult to find a place that is affordable and is suitable. I think a lot of students have to resort to places that are not very good, just because of how expensive the rent is”*. The survey indicated that students who lived in their preferred choice of accommodation had significantly higher wellbeing (PWI) scores than those who did not (*M* = 51.0, *SD* = 13.0, versus *M* = 45.3, *SD* = 13.8, *F* = 12.43, *p* < .001). As described in Table 3, one of the strongest predictors of international students’ wellbeing was living in their preferred accommodation setting (3.2% PWI variance, *p* < .001).

Exposure to a new culture in Australia and distance from familiar family and friend networks brought challenges such as loneliness for some, which together with having unsafe accommodation, impacted on students’ wellbeing. For example, one focus group participant noted: *“If you are feeling alone and you are not feeling safe, it impacts everything from top to bottom”.* Many students were acutely aware of their families’ worries about their safety: *“We’re scared all the time and so are our parents and our family. I live in a granny flat because my parents thought it would be safer for me to live close to another family that could do something if [something] danger[ous] happens”.* For some the limited sense of belonging and even discrimination reinforced lack of community and loneliness as one participant noted: *“I feel like I don’t feel a sense of belonging. This is one of my biggest feelings. I was actually discriminated against once.[this] contributed to my lack of sense of belonging”*. Conversely, participating in organised activities provided some students with a sense of community. For example, one participant said: *“I think one of the things that makes me feel very attached is that we have a church group in our student residence… They organised people to play [sport] together twice a week, which made me feel like I belonged to this community”.* Having a strong sense of religion or spirituality was also important to some students’ wellbeing and provided comfort in times of distress, and one student noted: “*I’m a very religious person, so whenever I am depressed, I pray and worship. I think that it helps me in reducing stress”.*

Students were asked in the survey to provide examples of how they engaged with the 5 Ways to Wellbeing, and key themes are noted here. One strong predictor of their wellbeing (as noted in Table 3) was by regularly helping others (as measured by the 5 Ways to Wellbeing, which contributed 2.3% PWI variance, *p* = .002). Frequently observed examples of ‘helping others’ included sharing skills and knowledge to help others (including volunteering), giving people their time, being a good listener, providing advice and encouragement, and checking in on family and friends. Regular social connection (1.1% PWI variance, *p =* .024) and physical activity (1.2% PWI variance, *p* = .018) were also strong predictors of high PWI scores. Frequently observed examples of ‘social connection’ included talking and listening to others (face-to-face, telephone or video calls, with telephone and video calls applying more during times of COVID-19 restrictions) and sharing experiences with others at home, in activities or through social events. Frequently observed examples of ‘being active’ included (either individuals or as a social activity) walking, jogging, gym, yoga, dancing, and group sports. Frequently observed examples of ‘learning new things’ included participating in online tutorials and classes, trying new hobbies, seeking new adventures, and meeting new people. It contributed 0.4% to PWI variance, however when controlling for all other variables, this contribution was not statistically significant (*p* > .05).

Uncertainty about the future, amplified during the COVID-19 pandemic, created additional pressure and stress for students. One student noted: *“I think a future aspect, which is somewhat relevant to everyone’s current profile while they are physically well, emotionally and mentally well, they keep on thinking about the future, how things are going to turn out in the future… so I think having that future sense of things in everything which we are doing [is important].* Mindfulness was suggested by some as an antidote to future based fears: *“So, for me, if thinking of the present, you are not stressed about what whatever is going to happen… like be optimistic and live in the present”.* Examples of ‘being mindful’ included meditation, prayer, mindfulness, practicing gratitude and yoga. When controlling for the effects of other variables, being mindful had no significant contribution to the variance in PWI (0.2%, *p* > .05) (0.2%, *p* > .05).

In terms of accessing support for mental health, international students noted significant barriers in the focus groups, including cost, language and cultural barriers, lack of information, stigma, and that it was their understanding that to access support one needed to have a ‘serious’ mental illness (i.e., struggling to cope with stress or anxiety was not a cause for seeking support). Furthermore, coping with stress and anxiety was something that needed to be ‘fixed’ at the individual level (or through engagement with family/friends, participating in exercise or in other activities), rather than through seeking professional help. For example, one student noted: *“For me, I usually solve psychological problems by exercising or talking to my friends”;* and another one said: “*I mostly seek help from family and friends, and they do help me a lot…However, it only works in the short term, not the long run”*. In fact, seeking help from professionals were often a source of concern for families as one student noted: “*When I talk to my family or friends back home, they don’t understand what mental health is. So sometimes I feel like if I want to talk about this, and if I want to discuss my mental health, I can’t do it with my friends and family back home because, for example, when I was speaking with a counsellor, I told my mum, and she freaked out”.* Negative views on accessing mental health services contributed 1.5% to PWI variance (*p* = 0.10).

Finally, it can be noted that both the survey and the focus groups also acted as vehicles for raising awareness around wellbeing. More than half of the survey respondents (*n* = 139, 57%) reported that completing the survey had been useful in prompting them to think about their health and wellbeing.

**Table 3:** Predictors of wellbeing (PWI)

	t	*p*-value	B	Part correlations	% variance in PWI explained by IV
Country of Birth					
China	2.42	0.016	− 0.143	0.115	1.3%
India	2.42	0.016	0.126	0.115	1.3%
Nepal	-2.35	0.019	− 0.133	− 0.112	1.25%
All other countries	1.49	0.138	0.090	0.076	0.58%
Living in preferred accommodation	3.77	< 0.001	− 0.184	− 0.178	3.2%
The cost of accommodation negatively impacts wellbeing	-1.86	0.064	− 0.095	− 0.088	0.77%
The people I live with negatively affects my health and wellbeing	-1.23	0.218	− 0.066	− 0.059	0.35%
I don’t feel safe in my current accommodation	1.15	0.250	0.061	0.055	0.30%
Language barriers prevent access to mental health services	0.461	0.645	0.025	0.022	0.04%
Negative views prevent access to mental health services	-2.58	0.010	− 0.138	− 0.123	1.5%
Five Ways					
Social connection	-2.27	0.024	− 0.119	− 0.108	1.1%
Being active	-2.38	0.018	− 0.128	− 0.113	1.2%
Being mindful	-1.02	0.311	− 0.055	− 0.048	0.2%
Helping others	-3.16	0.002	− 0.172	− 0.150	2.3%
Learning new things	-0.26	0.796	− 0.014	− 0.012	0.4%

## Discussion

Mental health has been defined as a complete state of subjective wellbeing as well as the absence of common mental disorders [43]. While we did not measure stress, mood, diagnosable mental health conditions, or other factors which contribute to wellbeing [9], the low subjective wellbeing scores of international students in this study indicate that they are far from flourishing. Others have found that international students in general, including Australian international students, are at increased risk for poor mental health outcomes [2, 3, 5].

The overall subjective wellbeing for international students in this sample (measured through the PWI), was substantially lower than that observed in the general Australian population and age-related Australian cohorts [32], and even cultural comparative norms [40–42]. It highlights that for these young people the quality of their wellbeing is much reduced, that many are having a tough time and that they are struggling to cope, particularly in relation to community connectedness and feeling secure about their future (which were scored the lowest). Both of these, and subjective wellbeing in general, might have been exacerbated during the COVID-19 pandemic which included periods of enforced isolation and other social distancing requirements. During this time international students were particularly at risk of isolation, uncertainty about their future, financial hardship, and homelessness, and many received inadequate support [44]. As this study was conducted in Victoria, Australia, generalisability to other international student cohorts is limited. Future research to replicate this study in non-pandemic times, particularly in relation to Australian international students’ subjective wellbeing, is recommended.

Similar to previous research [3, 23], this study found that a key factor impacting on wellbeing for international students was accommodation. Safe accommodation was important, and students (and their families) spent considerable time in advance researching locations with low crime rates and good public transport access. Inner city/suburb locations, secure access housing (e.g., apartments) as well as living close to friends/family were deemed safer options. Affordability of accommodation impacted negatively on wellbeing for two thirds of students and high costs of accommodation necessitated choices on shared housing/rooms, as also found by others [45]. For the majority shared housing/rooms also negatively impacted on their wellbeing, a finding echoed by others [23]. Overall, the study found that the strongest predictor of international students’ wellbeing was living in their preferred accommodation setting, as well as regularly helping others, being physically active and having regular social connection. Like others [10, 11], the study’s qualitative data also highlight the negative impact of loneliness, isolation and lack of belonging on international students’ wellbeing, and the great need for culturally appropriate opportunities for connection intentionally designed to enhance belonging and relational wellbeing [15, 16, 18, 22]. Spirituality and religion were a source of comfort for international students during times of distress. New Zealand research also identified significant correlations between religion/spirituality and psychological and social quality of life for international students [46], and that more so than for European students, religious coping strategies for Asian students was particularly helpful in improving psychological and social quality of life [47]. Thus, ongoing opportunities to identify where this can be incorporated to improve the psychological and social wellbeing of international students, are important.

In terms of help-seeking, international students noted a number of barriers to accessing support for mental health: cost of accessing support, language and cultural barriers, lack of information on where to find support and stigma. There was also a perception that to access support for mental health one needed to have a ‘serious’ mental illness, and that one should be able to cope with stress and anxiety by oneself, or with help from family or friends, rather than through professional help. These findings about barriers to accessing mental support in international students echoes those by other researchers [2, 3, 5]. Furthermore, our study shows that for those students who reported negative attitudes as a barrier to accessing mental health support, their subjective wellbeing was significantly lower.

In terms of study limitations, this survey and focus group-based research include the usual limitations of self-report data [30]. A further limitation of the study is that not all aspects relating to subjective wellbeing (e.g., autonomy, competence, relatedness, resilience, balance, interplay between different systems etc.) were measured, as this was beyond the study scope. Thus, the variance accounted for in terms of subjective wellbeing by the variables in Table 3 was approximately 16%. In particular the study highlights the importance of considering the impact of preferred accommodation (3.2%) on wellbeing. Future research could explore subjective wellbeing in relation to a wider array of variables; however, we would also encourage future researchers to include a focus on preferred accommodation for international students in measuring wellbeing.

While the recruitment methods were pragmatic and appropriate for this hard to reach population, the recruitment methods may have resulted in students who are not connected to student associations being under-represented in this study.

The relevance of the questions and measures used was enhanced by the co-design of the survey tool and focus groups. In terms of representativeness, by directly working through student associations who only represent international students the representativeness was enhanced. Cross checking themes of the focus groups with students further enhanced the reliability of the data.

## Conclusions

Beyond the sphere of academic support for international students [48], the findings of the study have implications for shaping policy and practice in service and facility provision around wellbeing, connectedness and help-seeking for mental health support. It highlights the importance of enabling and ensuring the rights and protections for international students, particularly in regard to safe and affordable accommodation. The ‘5 Ways to Wellbeing’ highlight how students can be encouraged to engage in their health and wellbeing. The study emphasises that to enhance the health and wellbeing of international students it is important to provide opportunities to connect with others, with students outside their culture and language group, with non-international students and with the wider Australian community. Promoting student-led social activities for international students and facilitating peer support networks through international students’ associations is one way to achieve this. There is also an important role for universities and tertiary education providers to play in facilitating this through integrated spaces and structures (including cultural/religious/spiritual spaces/structures), while also making available support and affordable mental health services and normalising help-seeking.

### Electronic supplementary material

Below is the link to the electronic supplementary material.

Supplementary Material 1

Supplementary Material 2

Supplementary Material 3

## Data Availability

The datasets used and/or analysed during the current study are available from the corresponding author on reasonable request.
