# Supplementary material for: ‘If you are feeling alone and you are not feeling safe, it impacts everything’: a mixed-methods exploration of international students’ accommodation, subjective wellbeing and mental health help-seeking
Source: BMC Public Health. 2024 May 8;24:1262. doi: 10.1186/s12889-024-18691-8 (PMC11077825; doi:10.1186/s12889-024-18691-8)
Supplement: Supplementary file 1 — Supplementary Material 1 [file 12889_2024_18691_MOESM1_ESM.docx]

**‘If you are feeling alone and you are not feeling safe, it impacts everything’: a mixed-methods exploration of international students' accommodation, subjective wellbeing and mental health help-seeking**

**FOCUS GROUP QUESTIONS**

**Introduction to Health and Wellbeing**

Today we’ll be exploring ideas around health, mental health and wellbeing for international students and the relationship to student housing. As we begin, we’d like to start with some definitions of what each of these are.

- The WHO defines health as:

“… a state of complete physical, mental and social well-being and not merely the absence of disease or infirmity”.

- Beyond Blue states that mental health is:

“… about being cognitively, emotionally and socially healthy – the way we think, feel and develop relationships - and not merely the absence of a mental health condition”.

- The Personal Wellbeing Index considers subjective wellbeing to include:

“… standard of living, health, achieving in life, relationships, safety, community-connectedness, and future security”.

**Question about Health and Wellbeing**

- Do you agree with these statements?
- Is there anything you would add to the definitions of health and wellbeing?
- Do you find it easy to access health and wellbeing support services?
- If not, what are some of the barriers to access, e.g., language, cultural, cost, location, other?

**Housing**

- As a student, what type of accommodation do you prefer to live in? Explain
- Does your accommodation/type of house that you live in effect your health and wellbeing? Explain. (Positive and negatives)
- Does where you live (the area/suburb) effect your health and wellbeing? Explain
- Does who you live with/living with other people (or on your own) effect your health and wellbeing? Explain
- Does the cost of rent effect your health and wellbeing? Explain

**Safety**

- Is feeling safe important to your health and wellbeing?
- What makes you feel safe in your accommodation?
- What makes you feel unsafe in your accommodation?
- Survey results suggest students feel least safe in high-rise apartments and at Unilodge – why do you think that is?

**5 Ways to Wellbeing**

Do you know about the ‘5 Ways to Wellbeing’? Do you think they can improve wellbeing?

1. Connecting with others

2. Being active and exercising

3. Learning new things

4. Being aware and mindful

5. Helping others

**Outcome/recommendation questions:**

Our survey told us that walking was the most common exercise for international students and that connecting with others and a local community is important to health and wellbeing.

- - If there was a walking group activity organised once a week in your area with other international students would you join? Why, why not?
  - What if the walking group also had a focus beyond social connection with others, such as learning local history? Or mentoring? Or mindfulness and mediation? Would that be of interest to you?
  - Who would be best to organise and promote this walking and learning activity? – student club, university, local council, study Melbourne, community group?
  - What would enable you to participate and what would be a barrier to that?

**Community**

Our survey told us that people who had low life satisfaction, didn’t feel as though they belonged to a community.

- - What does belonging to a community mean to you?
  - What does it look like? (Clarify what community is – local, global or cultural?)
  - When do you feel like you belong, rather than just knowing people?
  - Is your educational institution a place where you feel like you belong?
