# Supplementary material for: ‘If you are feeling alone and you are not feeling safe, it impacts everything’: a mixed-methods exploration of international students’ accommodation, subjective wellbeing and mental health help-seeking
Source: BMC Public Health. 2024 May 8;24:1262. doi: 10.1186/s12889-024-18691-8 (PMC11077825; doi:10.1186/s12889-024-18691-8)
Supplement: Supplementary file 2 — Supplementary Material 2 [file 12889_2024_18691_MOESM2_ESM.docx]

**‘If you are feeling alone and you are not feeling safe, it impacts everything’: a mixed-methods exploration of international students' accommodation, subjective wellbeing and mental health help-seeking**

**One-Way ANOVA Tests**

|  | *p*-value | F-value | Effect size |
| --- | --- | --- | --- |
| **Demographics** |  |  |  |
| Gender | .591 | .527 | 0.003 |
| Country of Birth | <.001* | 5.11 | 0.001 |
| China |  |  |  |
| India |  |  |  |
| Nepal |  |  |  |
| Other South-East Asian Country |  |  |  |
| Other Country |  |  |  |
| Level of education (current enrolment/recently completed) | .101 | 1.95 | .021 |
| TAFE |  |  |  |
| University (undergraduate) |  |  |  |
| University (postgraduate) |  |  |  |
| English language course/other (e.g., VET, private institution) |  |  |  |
| Language | .913 | .012 | .006 |
| **Living situation** |  |  |  |
| Accommodation (preferred) | <.001* | 12.43 | .074 |
| Other People in household | .631 | .232 | .013 |
| Recreation space in accommodation | .612 | .257 | .048 |
| Did you know people in your household prior to moving in? | .189 | 1.734 | .044 |
| **Impact of accommodation** |  |  |  |
| The cost of my accommodation negatively affects my wellbeing | .011* | 4.56 | .024 |
| The people I live with negatively affects my health and wellbeing | .020* | 3.93 | .021 |
| I don’t feel safe in my current accommodation | .004* | 5.544 | .017 |
| **Barriers to services** |  |  |  |
| Language barriers or lack of culturally appropriate services | .039* | 3.62 | .017 |
| Cost of services | 1.530 | .218 | .008 |
| Negative views (stigma) | <.001 | 8.17 | .042 |
| Time constraints | .603 | .507 | .003 |
| Lack of information | .320 | 1.14 | .006 |
|  |  |  |  |
