# Supplementary material for: ‘If you are feeling alone and you are not feeling safe, it impacts everything’: a mixed-methods exploration of international students’ accommodation, subjective wellbeing and mental health help-seeking
Source: BMC Public Health. 2024 May 8;24:1262. doi: 10.1186/s12889-024-18691-8 (PMC11077825; doi:10.1186/s12889-024-18691-8)
Supplement: Supplementary file 3 — Supplementary Material 3 [file 12889_2024_18691_MOESM3_ESM.pdf]

Positive International Student Residences
This survey is part of a study researching student health and wellbeing in relation to student accommodation in Australia.

This survey is for international students, 18 years of age or older, who are, or have recently been, a resident in Victoria while studying at a university or tertiary institution.

This project is being conducted by Associate Professor Tim Corney and Dr Catherine Lou from the Institute for Sustainable Industries and Livable Cities at Victoria University. This project is funded by Study Melbourne's International Student Welfare Program, a Victorian Government initiative.

The survey is anonymous and response information will be used for research purposes only.

The survey will take approximately 15 minutes to complete. For further information, please contact: wellbeing.research@vu.edu.au.

As an international student, completing this survey will provide useful feedback to help meet the needs of other international students.

本调查有普通话版本。请点击此处完成中文版本的调查问卷。

Definitions

This survey considers the health and wellbeing of international students living in Australia. This survey will adopt broad definitions of health and mental health.

The World Health Organisation defines **health** as "… a state of complete physical, mental and social well-being and not merely the absence of disease or infirmity".

The organisation Beyond Blue suggests that **mental health** "… is about being cognitively, emotionally and socially healthy – the way we think, feel and develop relationships - and not merely the absence of a mental health condition".

Support

While it is not the intention of this survey, participants may find some questions uncomfortable. If you do not wish to answer a question, you may skip it and go to the next question or stop the survey immediately. If you become distressed while participating in this survey, please contact the services listed here.

Consent to participate

This is an online survey, so there is no separate consent form. When you received the link to this survey, you were provided with the document '*Information for Participants*', explaining the project, how your information will be used and how your privacy will be protected. Please review that information (or view it again by following the link here).

Having read this information, and accepted the invitation to proceed to the survey, this indicates that **you are consenting to participate in the research**. Please click on the button below to proceed or exit the survey.

- ◯ I consent
- ◯ I do not consent (ticking this box will exit the survey)

How old are you?

- ◯ Under 18 years old
- ◯ 18-25 years old
- ◯ Older than 25 years

Are you a current student enrolled in a university or tertiary institution based in Australia or have you recently completed your studies at a university or tertiary institution based in Australia?

- ◯ Yes, I am a current student and I currently or usually live in Australia.
- ◯ Yes, I am a current student but I have not lived in Australia.
- ◯ Yes, I have recently completed my studies and I lived / am living in Australia.
- ◯ None of the above.

Personal Wellbeing Index

The following questions come from the *Personal Wellbeing Index,* which measures subjective wellbeing through questions of satisfaction in specific life domains. These life domains include: standard of living, health, achieving in life, relationships, safety, community-connectedness, and future security.

The Personal Wellbeing Index was developed by the Australian Centre on Quality of Life (*International Wellbeing Group (2013). Personal Wellbeing Index: 5th Edition. Melbourne: Australian Centre on Quality of Life, Deakin University http://www.acqol.com.au/instruments#measures).*

These questions are directed to people's feelings about themselves. As you answer the questions, just think of the question you have been asked in the way it makes sense to you. There is no right or wrong answer.

The following questions ask how *satisfied* you feel, on a scale from zero to 10. **Zero** means you feel no satisfaction at all and **10** means you feel completely satisfied.

Thinking about your own life and personal circumstances, how satisfied are you **with your life as a whole?**

No satisfaction at all - 0	1	2	3	4	5	6	7	8	9	Completely satisfied - 10
○	○	○	○	○	○	○	○	○	○	○

How satisfied are you with:

	No satisfaction at all 0	1	2	3	4	5	6	7	8	9	Completely satisfied 10
your standard of living?	○	○	○	○	○	○	○	○	○	○	○
your health?	○	○	○	○	○	○	○	○	○	○	○
what you are achieving in life?	○	○	○	○	○	○	○	○	○	○	○
your personal relationships?	○	○	○	○	○	○	○	○	○	○	○
how safe you feel?	○	○	○	○	○	○	○	○	○	○	○
feeling part of your community?	○	○	○	○	○	○	○	○	○	○	○
your future security?	○	○	○	○	○	○	○	○	○	○	○

(OPTIONAL) How satisfied are you with **your spirituality** or **religion?**

No satisfaction at all - 0	1	2	3	4	5	6	7	8	9	Completely satisfied - 10
○	○	○	○	○	○	○	○	○	○	○

5 ways - Connecting and Wellbeing

Five Ways to Wellbeing

This next section introduces you to five simple and effective ways to improve your mental health and wellbeing. The five ways are connecting, being active, learning, being aware and helping others.

You can learn more about the Five Ways to Wellbeing **here**.

This research is about your experience as an international student in Australia. As you answer these questions, please consider how you previously did, or will do

these things in a post-COVID-19 world, without lockdowns and restrictions.

The following questions will ask you to think about how you **connect** with others. These questions ask you to think about your social life and your relationships with others around you.

Developing close relationships and socialising with friends, family and others, is important for good health and wellbeing. Broadening your social networks and range of relationships with others in the wider community, is also important for your wellbeing.

Remember, we're interested in what ways you *usually* connect with others – it might be helpful to consider what you did before COVID-19.

Please rate the importance of **connecting socially** with other people to your wellbeing?

Extremely important	Very important	Moderately important	Slightly important	Not at all important
○	○	○	○	○

In what ways do you **connect** with other people? (e.g. talk, listen, be there for others, do things with others.)

How regularly do you **connect** with others?

Daily	4-6 times a week	2-3 times a week	Once a week	Rarely	Never
○	○	○	○	○	○

What ideas do you have that would enable people to **connect** more often? (e.g. things *you* could do or things *organisations* (like universities, community groups or student groups) could do.)

5 ways - Being Active and Wellbeing

The following questions are about your **physical activity**. These questions ask you to think about how you are being physically active in organized / formal or informal ways.

Being active is important for good health, to help keep your mind and body working well. Doing something every day can help make you feel good and clear your mind!

Remember, we're interested in what ways you are *usually* active – it might be helpful to consider what you did before COVID-19.

Please rate the importance of **being active** to your wellbeing?

Extremely important	Very important	Moderately important	Slightly important	Not at all important

○ ○ ○ ○ ○

In what ways are you **active**? (e.g. walking, running, dancing or gardening – whatever keeps you moving regularly.)

How regularly are you **active**?

Daily	4-6 times a week	2-3 times a week	Once a week	Rarely	Never
○	○	○	○	○	○

What ideas do you have that would enable people to **be active** more often? (e.g. things *you* could do or things *organisations* (like universities, community groups or student groups) could do.)

5 ways - Learning and Wellbeing

The following questions will ask you to think about **learning new things**. These questions ask you to think about learning and interests outside of your formal study.

Learning new things is associated with higher levels of mental health and wellbeing. Learning can mean you try something new, take up a hobby or rediscover an old interest.

Remember, we're interested in what ways you *usually* learn new things – it might be helpful to consider what you did before COVID-19.

Please rate the importance of **learning new things** and/or **having an interest** (outside of your formal study) to your wellbeing?

Extremely important	Very important	Moderately important	Slightly important	Not at all important
○	○	○	○	○

In what ways do you **learn new things**? (e.g. try a new hobby, seek out new experiences or set yourself a challenge.)

How regularly do you **learn new things** or participate in an **interest** outside of your formal study?

Daily	4-6 times a week	2-3 times a week	Once a week	Rarely	Never
○	○	○	○	○	○

What ideas do you have that would enable people to **learn new things** more often? (e.g. things *you* could do or things *organisations* (like universities, community groups or student groups) could do.)

5 ways - Awareness and Wellbeing

The following questions will ask you to think about the ways that you are **mindful** and **notice and appreciate things around you**. Notice and appreciate new sights, smells, and sounds as you do your daily activities. Take notice of what you are feeling. Focusing on the 'here and now' can help you feel calm and reduce stress.

Remember, we're interested in what ways you are *usually* mindful – it might be helpful to consider what you did before COVID-19.

Please rate the importance of **being mindful** or **noticing and appreciating things around you** to your wellbeing?

Extremely important	Very important	Moderately important	Slightly important	Not at all important
○	○	○	○	○

In what ways do you practice being **mindful** or **noticing and appreciating things around you**?(e.g. being in the moment, savouring the things you enjoy or noticing the change of seasons.)

How regularly do you practice **being mindful** or **noticing and appreciating things around you**?

Daily	4-6 times a week	2-3 times a week	Once a week	Rarely	Never
○	○	○	○	○	○

What ideas do you have that would enable people to be **mindful** or **notice and appreciate things around you** more often? (e.g. things *you* could do or things *organisations* (like universities, community groups or student groups) could do.)

5 ways - Helping Others and Wellbeing

The following questions will ask you to think about **helping others**. These questions ask you to think about ways you help others in formal ways (for example, through volunteering) or informally (for example, through random acts of kindness).

Helping others is associated with higher levels of mental health and wellbeing. Do something nice for a friend, or a stranger, including thanking someone, smiling, volunteering your time or joining a community group. Seeing yourself, and your happiness, linked to the wider community can be rewarding and creates connections with the people around you.

Remember, we're interested in what ways you *usually* help others – it might be

helpful to consider what you did before COVID-19.

Please rate the importance of **helping others** to your wellbeing?

Extremely important	Very important	Moderately important	Slightly important	Not at all important
○	○	○	○	○

In what ways do you **help others**? (e.g. giving your time, your words, your presence.)

How regularly do you **help others**?

Daily	4-6 times a week	2-3 times a week	Once a week	Rarely	Never
○	○	○	○	○	○

What ideas do you have that would enable people to **help others** more often? (e.g. things *you* could do or things *organisations* (like universities, community groups or student groups) could do.)

Mental Health and Wellbeing

The next section of the survey asks about your experience of wellbeing. It will ask some questions about your mental wellbeing. If you find a question distressing, stop and move to the next question, or exit the survey and please contact one of the services listed here.

Please think about whether you agree or disagree with the following statements:

	Strongly agree	Somewhat agree	Neither agree nor disagree	Somewhat disagree	Strongly disagree
Language barriers or lack of culturally appropriate services restrict me from accessing health and wellbeing support.	○	○	○	○	○
The cost of services restricts me from accessing health and wellbeing support.	○	○	○	○	○

	Strongly agree	Somewhat agree	Neither agree nor disagree	Somewhat disagree	Strongly disagree
Other people's negative views about mental health restrict me from accessing health and wellbeing support.	○	○	○	○	○
Time constraints (for example, long waiting times or being too busy with work or study commitments) restrict me from accessing health and wellbeing support.	○	○	○	○	○
A lack of information (for example, being unsure of where to go or what services I need) restricts me from accessing health and wellbeing support.	○	○	○	○	○

Accommodation Demographics

Accommodation

This survey is interested in the relationship, if any, of accommodation to wellbeing.

We are interested in your **current**, or **most recent**, accommodation as a **student** living in Australia.

What type of accommodation in Australia do you usually live in?

- ○ UniLodge or Student Village or University college/hall of residence
- ○ Flat/Unit
- ○ High-rise apartment
- ○ House
- ○ Other, please specify: ____________

Is this your preferred choice of accommodation?

Yes	No
○	○

Does your accommodation have recreation or outdoor areas **within** the accommodation itself? This might include a gym, garden or shared outdoor space.

Yes	No
○	○

Are you living with other people in your accommodation?

- ◯ No, I live on my own.

- ◯ Yes, I live with other people but I have my own room.
- ◯ Yes, I live with other people and I share a room with one other person.
- ◯ Yes, I live with other people and I share a room with two or more people.

Did you know the people you live with **before** you moved in?

Yes	No
◯	◯

How much rent do you pay per week in $AUD dollars?

I don't pay rent	below $150 AUD	$150-199 AUD	$200-249 AUD	$250-299 AUD	above $300 AUD
◯	◯	◯	◯	◯	◯

Accommodation (wellbeing and satisfaction)

Please think about whether you agree or disagree with the following statements:

	Strongly agree	Somewhat agree	Neither agree nor disagree	Somewhat disagree	Strongly disagree
The cost of my accommodation negatively affects my wellbeing.	◯	◯	◯	◯	◯
The people I live with negatively affect my health and wellbeing.	◯	◯	◯	◯	◯
I don't feel safe in my current accommodation and that affects my health and wellbeing.	◯	◯	◯	◯	◯

Please explain how your current accommodation affects your health and wellbeing:

Demographics

The following are some demographics questions. These final demographics questions help us to understand you better.

What level is your current or recently completed course of study?

- ◯ TAFE (certificate or diploma)
- ◯ University (undergraduate)
- ◯ University (postgraduate)
- ◯
- ◯

English Language Intensive Course for Overseas Students (ELICOS) College

Other (please specify)

How many years have you been living in Australia? (Please choose the nearest year):

- ○ Less than 1 year
- ○ 1 year
- ○ 2 years
- ○ 3 years
- ○ 4 years or more

How do you identify in terms of gender?

- ○ Male
- ○ Female
- ○ Non-binary or gender diverse
- ○ Transgender
- ○ Agender
- ○ Prefer not to say
- ○ Other, please specify: ________

What is your Nationality or Country/Region of Citizenship?

[dropdown]

What is the language you speak at home?

[dropdown]

What is the language you speak at home?

____

What is/was the postcode of your most recent student accommodation in Australia? Please enter the four digit postcode.

Postcode ________

How did you hear about this survey?

- ○ Friend forwarded it to me
- ○ Council of International Students Australia
- ○ Australian Federation of International Students
- ○ Victoria Chinese Student & Scholar Association
- ○ Victoria University International Student Association
- ○ Victoria University Chinese Students Scholars Association
- ○ Council of Australian Postgraduate Associations (CAPA)
- ○ Other, please specify ________

Have you accessed the services of Study Melbourne?

Conclusion

Yes

○

No, I have heard of Study Melbourne but I have not accessed their services

No, I don't know about Study Melbourne. ○

Has this survey been useful for thinking about your health and wellbeing?

Extremely useful	Very useful	Moderately useful	Slightly useful	Not at all useful
○	○	○	○	○

Thank you for participating in this survey. The findings from this research project will help improve the experience of international students and their health and wellbeing outcomes.

Study Melbourne have lots of resources to support you as an international student. You can find their website here.

VICTORIA UNIVERSITY

STUDY MELBOURNE

Powered by Qualtrics
